# Heart failure with preserved ejection fraction and atrial fibrillation: recent advances and open questions

**DOI:** 10.1186/s12916-023-02764-3

**Published:** 2023-02-13

**Authors:** Laurent Fauchier, Arnaud Bisson, Alexandre Bodin

**Affiliations:** Service de Cardiologie, Centre Hospitalier Universitaire Trousseau Et Faculté de Médecine, Université François Rabelais, 37044 Tours, France

**Keywords:** Heart failure with preserved ejection fraction, Atrial fibrillation, SGLT2 inhibitors

## Abstract

Atrial fibrillation (AF) and heart failure (HF) are frequently associated and can be caused or exacerbated by each other through different mechanisms. AF is particularly common in patients with heart failure with preserved ejection fraction (HFpEF) defined as left ventricular ejection fraction (LVEF) ≥ 50%, with a prevalence ranging around 40–60%.

In two recent trials, treatment with SGLT2 inhibitors resulted in a lower risk of worsening heart failure or cardiovascular death than placebo in patients with HFpEF, and SGLT2 inhibitors similarly improved prognosis whether patients had AF or not at enrolment. Analyses for subgroups of interest of patients with HFpEF likely to be at higher risk of AF (particularly those with older age or obesity) similarly indicated a consistent benefit with SGLT2 inhibitors. That subgroup in patients with HFpEF is those with a history of previous HF with LVEF ≤ 40%. The EAST-AFNET 4 trial indicated that early rhythm-control therapy was associated with a lower risk of adverse cardiovascular outcomes than usual care among patients with recent AF and cardiovascular conditions, including those with HF. In patients with AF and HF included in the CABANA trial, catheter ablation produced marked improvements in survival, freedom from AF recurrence, and quality of life compared to drug therapy. When strategies aiming at rhythm control eventually fail in patients with AF and HFpEF, a strategy of rate control with atrioventricular junction ablation and cardiac resynchronisation should be discussed since it may also reduce all-cause mortality.

Finally, and in conclusion, considering that patients with AF and HFpEF may have a variety of cardiovascular and non-cardiovascular additional comorbidities, they are among those likely to have the highest clinical benefit being adherent to a holistic and integrated care management of AF following the ABC (Atrial Fibrillation Better Care) pathway.

## Background

Atrial fibrillation (AF) and heart failure (HF) are frequently associated and can be caused or exacerbated by each other through different mechanisms including cardiac remodelling and rate-related left ventricular incompetency [1–3]. AF is particularly common in patients with heart failure with preserved ejection fraction (HFpEF, defined as left ventricular ejection fraction [LVEF] ≥ 50%), with a prevalence ranging around 40–60% [4, 5]. AF and HFpEF may manifest with similar symptoms, and diagnostic uncertainties may exist for the diagnosis of HFpEF due to their interrelations influencing test results for echocardiography and natriuretic peptides [2]. HF patients with AF have a poorer prognosis than those with sinus rhythm and, importantly, the higher risk brought by AF is generally higher in patients with HFpEF than in those with HF and a reduced ejection fraction (HFrEF defined as LVEF ≤ 40%) [4]. This commentary discusses some recent advances in the understanding for the natural history of patients with HFpEF associated with AF and for the several different aspects of their medical management.

## How does AF pattern affect prognosis in HFpEF?

In unselected patients with AF, those with permanent AF are more likely to be older and to have HF than those with paroxysmal AF [6]. By contrast, patients with paroxysmal AF may have a higher prevalence of coronary artery disease [7]. The rates of death, stroke, and worsening HF are generally higher in patients with persistent and permanent AF than in patients with paroxysmal AF [6]. Progression from paroxysmal to persistent/permanent AF is also associated with adverse cardiovascular events, hospitalisations, and death [1]. The picture is a bit different when AF is associated with HF. Although paroxysmal AF is often characterised by lower atrial structural remodelling or less severe atrial cardiomyopathy when compared to non-paroxysmal AF, patients with HF and paroxysmal AF may have a higher crude and adjusted risk of HF hospitalisation [7]. This has also been reported recently in patients with HFpEF [8]. Why paroxysmal (versus non-permanent) AF is associated with a higher risk is uncertain. It is possible that episodes of paroxysmal of AF reflect HF instability (e.g. rises in atrial pressure triggering together episodes of AF and decompensation leading to hospital admission) or that acute changes in heart rhythm per se worsen HF in case of alternating fast ventricular rate due to AF with normal sinus rhythm. This may be particularly true for patients with HFpEF known to be easily decompensated in case of acute hemodynamic changes.

## Patients with HFpEF and history of previously reduced EF: was it AF and tachycardiomyopathy?

An interesting subgroup in patients with HFpEF is those with a history of previous HFrEF with LVEF ≤ 40%. Who are these patients with HFpEF and history of previously reduced LVEF? We think that two main reasons possibly overlapping may explain this profile. The first one is the setting where medical drugs indicated for HFpEF were able to improve LVEF, a scenario that may be seen for around 1/3 of patients with HFrEF (compared to grossly 1/3 with stable LVEF and 1/3 with worsening LVEF in spite of optimal drug therapy) [9]. The other possibility is that HFrEF was related to a transient or a curable cause that may include for example ischemic aetiology with efficient revascularisation or valve disease treated with surgery or percutaneous intervention [2, 9]. However, one of the most striking examples of HFrEF with complete recovery is cardiomyopathy directly and purely induced by persistent arrhythmias (so-called tachycardiomyopathy) among which AF is the most common cause [2, 9, 10]. When AF causes HF, the clinical course may be more favourable than with other causes of HF although patients may not have a complete healing and may shift from HFrEF to HFpEF. In contrast, the development of AF in patients with pre-existing HF (whether this is HFrEF or HFpEF) is frequently associated with a worse prognosis, including a higher risk of stroke and increased mortality [2, 11]. These elements should inspire future trials of specific therapeutics for HFpEF that would include the poorly evaluated population of patients with an improved LVEF, particularly when temporary AF has been involved in the development of transient HFrEF.

## Are there benefits of SGLT2 inhibitors when HFpEF is associated with AF?

Sodium–glucose cotransporter 2 (SGLT2) inhibitors, initially developed for the treatment of type 2 diabetes mellitus, have shown major clinical benefits for patients with HFrEF in the last years (with or without diabetes), and in the last months for those with HFpEF [12]. Two trials indeed evaluated empagliflozin and dapagliflozin in patients with heart failure and a left ventricular ejection fraction of more than 40%, with similar inclusion and exclusion criteria and a similar primary composite outcome. Treatment with SGLT2 inhibitors resulted in a lower risk of worsening heart failure (defined as hospitalisation or unexpected visit for heart failure) or cardiovascular death than placebo in the two trials [13, 14]. Dapagliflozin brought a significant clinical benefit in the subgroup of patients with HFpEF and history of previous HFrEF with LVEF ≤ 40% (with a numerically lower HR of the primary combined endpoint of 0.74 compared to 0.84 for the other group of patients) [14]. A major point to be mentioned is that SGLT2 inhibitors similarly improved prognosis whether patients had AF or not at enrolment [13–15]. The treatment effect for the composite endpoint of cardiovascular death or first hospitalisation for HF was indeed consistent for patients with AF (HR 0.77, 95% CI 0.69–0.87) and those with no AF (HR 0.83, 95% CI 0.72–0.95), and there was no statistical heterogeneity between empagliflozin and dapagliflozin in the subgroups of patients with AF [15]. Analyses for subgroups of interest of patients with HFpEF likely to be at higher risk of AF (particularly those with older age or obesity) similarly indicated a consistent benefit with SGLT2 inhibitors and no apparent heterogeneity between empagliflozin and dapagliflozin [15].

## Rhythm control therapy for all patients with atrial fibrillation and HFpEF?

The occurrence of paroxysmal AF may reflect deterioration in HF with congestion and higher atrial pressure precipitating both episodes of AF and decompensation of HF. Alternatively, the occurrence of paroxysmal AF related to electrical instability may lead to a sudden increase in ventricular rate with loss of atrial systole and may be the direct cause of decompensation [8]. If the latter is true, prevention of AF by rhythm control using an antiarrhythmic agent or catheter ablation might reduce the risk of HF decompensation.

The EAST-AFNET4 trial recently indicated that early rhythm-control therapy was associated with a lower risk of adverse cardiovascular outcomes than usual care among patients with recent AF (diagnosed within 1 year) and cardiovascular conditions [16]. This applied to patients with HF (*n* = 798), a majority of whom having HFpEF (56% of those with HF). An ancillary analysis has been presented for these patients [17]. The primary outcome (composite endpoint of death from cardiovascular causes, stroke, hospitalisation with worsening of HF or acute coronary syndrome) occurred in 94 of 396 HF patients randomly assigned to early rhythm control and in 130 of 402 HF patients randomly assigned to usual care (hazard ratio [HR] 0.74, 95% CI 0.56–0.97, *p* = 0.03). The treatment effect was not different from that in patients with normal left ventricular function and with no signs of HF (HR 0.81, 95% CI 0.66–1.0, *p* = 0.06; interaction *p* between treatment and HF = 0.63). Patients with HFpEF had a lower risk for the first primary outcome compared with those with HFrEF. However, the highest improvement in NYHA class occurred in patients with HFpEF.

## Drugs or catheter ablation for rhythm and rate control in AF with HFpEF?

Interestingly, exploratory analyses of AF patients in the EAST-AFNET 4 study suggested that treatment with amiodarone, but not treatment with flecainide, propafenone, or dronedarone, was potentially associated with early HF hospitalisations in patients with HFpEF [17]. This may be a surprising finding since amiodarone is considered a relatively safe antiarrhythmic drug in patients with HF [1]. It thus suggests that further clinical research is needed to define the optimal antiarrhythmic drug therapy in patients with HFpEF.

Previously available evidence of AF ablation in HFpEF until recently consisted of a few small observational reports. An ancillary analysis of the randomised CABANA trial reported outcomes with catheter ablation and antiarrhythmic drug therapy in 778 patients with AF and stable HF at baseline, the majority of whom (79%) having HFpEF [18]. Catheter ablation produced marked improvements in survival, freedom from AF recurrence, and quality of life compared to drug therapy. In the intention-to-treat analysis, the ablation arm had a significant 36% relative reduction in the primary composite endpoint of death, disabling stroke, serious bleeding, or cardiac arrest and a 43% relative reduction in all-cause mortality. These results tended to be better than in the group of patients with no HF, highlighting the possible benefit of AF ablation in case symptoms and functional impairment may be attributed to the combined effects of AF and HFpEF. However, the effects on HF hospitalisations were small and not significant and the authors concluded that the results should be reproduced in a confirmatory trial.

**Fig. 1:**
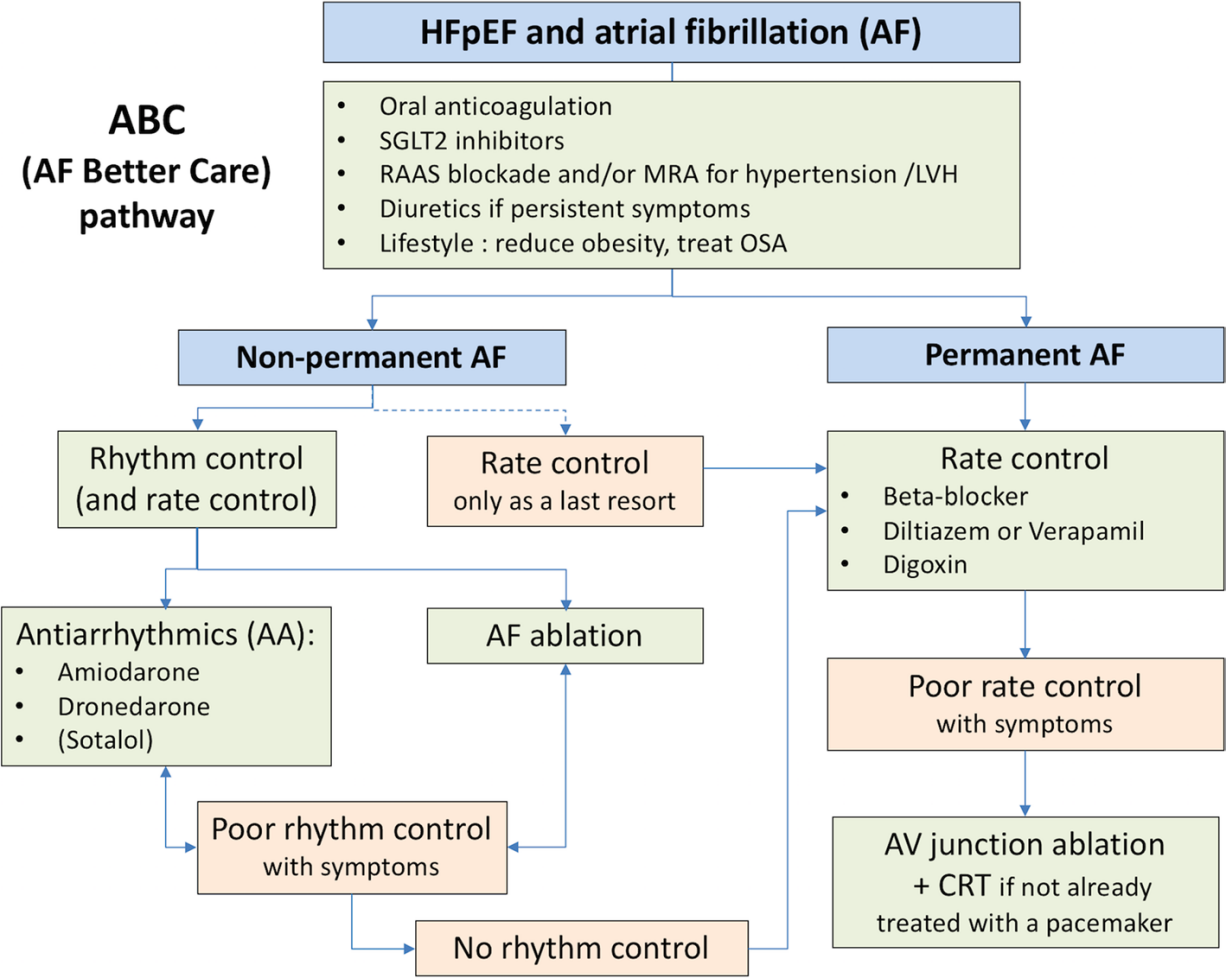
Principles for the holistic ABC approach including rate/rhythm control in AF management for patients with HFpEF

When strategies aiming at rhythm control eventually fail, a strategy of rate control with atrioventricular junction ablation and cardiac resynchronisation should be discussed in case of AF with HFpEF since it reduced all-cause mortality in the APAF-CRT trial for patients with permanent AF and narrow QRS hospitalised for HF, irrespective of their baseline EF [19] (Fig. 1).

## HFpEF: a setting where the holistic ABC approach is of major interest for the management of AF with HF

Oral anticoagulation is also a major pillar to improve outcomes in patients with AF and HFpEF, but we need to look beyond anticoagulation in these patients [1]. However, a simple unique and one-size-fits-all approach may not be applicable or sufficient in the case of HFpEF with AF. Regarding early rhythm control for possibly improving outcomes in AF patients, most benefit occurs if intervention is early and in younger patients and those with fewer coexisting conditions and if it also includes an association of care with attention to anticoagulation management, risk-factor control, lifestyle factors, and regular follow-up visits needed to ensure adherence and effective care approaches [20].

## Conclusions

Perhaps one of the most important messages is indeed that patients with AF and HFpEF may have a variety of cardiovascular and non-cardiovascular additional comorbidities. Clinical events are common, despite anticoagulation and other medical therapies needed for HF [1, 2]. There has been a move toward a more holistic approach to the management of AF, summed up as the ABC (Atrial Fibrillation Better Care) pathway: (A) avoidance of stroke with the use of anticoagulation; (B) better management of symptoms with patient-centred, symptom-directed decisions on rate or rhythm control; and (C) cardiovascular and coexisting-condition risk management, including attention to psychological factors and lifestyle [21]. Adherence to the ABC pathway is associated with better clinical outcomes, including lower risks of all-cause death and cardiovascular death, stroke, and hospitalisation for cardiovascular cause [22], which explains its inclusion in most recent guidelines [1]. Patients with AF and HFpEF are probably those who may have the highest clinical benefit being adherent to the integrated care management of AF following the ABC pathway.

## Data Availability

Not applicable.
